# GLI1 and AXIN2 Are Distinctive Markers of Human Calvarial Mesenchymal Stromal Cells in Nonsyndromic Craniosynostosis

**DOI:** 10.3390/ijms21124356

**Published:** 2020-06-19

**Authors:** Lorena Di Pietro, Marta Barba, Chiara Prampolini, Sabrina Ceccariglia, Paolo Frassanito, Alessia Vita, Enrico Guadagni, Davide Bonvissuto, Luca Massimi, Gianpiero Tamburrini, Ornella Parolini, Wanda Lattanzi

**Affiliations:** 1 Dipartimento Scienze della Vita e Sanità Pubblica, Università Cattolica del Sacro Cuore, 00168 Rome, Italy; lorena.dipietro@unicatt.it (L.D.P.); marta.barba@unicatt.it (M.B.); sabrina.ceccariglia@unicatt.it (S.C.); alessiavita89@gmail.com (A.V.); enrico.guadagni@unicatt.it (E.G.); ornella.parolini@unicatt.it (O.P.); 2 Fondazione Policlinico Universitario A. Gemelli IRCCS, 00168 Rome, Italy; paolo.frassanito@policlinicogemelli.it (P.F.); davide.bonvissuto@unicatt.it (D.B.); luca.massimi@policlinicogemelli.it (L.M.); gianpiero.tamburrini@unicatt.it (G.T.); 3 Dipartimento Testa-Collo e Organi di Senso, Università Cattolica del Sacro Cuore, 00168 Rome, Italy; chiara.prampolini@unicatt.it; 4 Dipartimento Neuroscienze, Università Cattolica del Sacro Cuore, 00168 Rome, Italy

**Keywords:** mesenchymal stromal cells, stem cell niche, GLI1, AXIN2, osteogenesis, bone development, nonsyndromic craniosynostosis, cranial suture, regenerative medicine, personalised medicine

## Abstract

All skeletal bones house osteogenic stem cell niches, in which mesenchymal stromal cells (MSC) provide progenitors for tissue growth and regeneration. They have been widely studied in long bones formed through endochondral ossification. Limited information is available on the composition of the osteogenic niche in flat bones (i.e., skull vault bones) that develop through direct membranous ossification. Craniosynostosis (CS) is a congenital craniofacial defect due to the excessive and premature ossification of skull vault sutures. This study aimed at analysing the expression of GLI1, AXIN2 and THY1 in the context of the human skull vault, using nonsyndromic forms of CS (NCS) as a model to test their functional implication in the aberrant osteogenic process. The expression of selected markers was studied in NCS patients’ calvarial bone specimens, to assess the in vivo location of cells, and in MSC isolated thereof. The marker expression profile was analysed during in vitro osteogenic differentiation to validate the functional implication. Our results show that GLI1 and AXIN2 are expressed in periosteal and endosteal locations within the osteogenic niche of human calvarial bones. Their expression is higher in MSC isolated from calvarial bones than in those isolated from long bones and tends to decrease upon osteogenic commitment and differentiation. In particular, AXIN2 expression was lower in cells isolated from prematurely fused sutures than in those derived from patent sutures of NCS patients. This suggests that AXIN2 could reasonably represent a marker for the stem cell population that undergoes depletion during the premature ossification process occurring in CS.

## 1. Introduction

Craniofacial bones are flat bones formed through intramembranous ossification, differently from most of the other bones of the human skeleton that develop through endochondral ossification [1]. Intramembranous bone ossification is indeed a direct process in which osteoblasts differentiate directly from mesenchymal cells, without the formation of cartilage precursors. 

In the craniofacial region, intramembranous bone development starts with the aggregation of mesenchymal stromal cells (MSC) into condensation centres, where they grow and proliferate, forming clusters [2]. Once the cluster reaches a critical size, MSC in the centre start to differentiate into osteoblasts. The growth of immature bone occurs at the osteogenic fronts, where cells actively proliferate. When an osteogenic front joins its neighbour, the two fronts either merge to create a single bone or give rise to a suture [3].

A suture is hence a fibrous joint composed of two osteogenic fronts and the interposed mesenchyme-derived fibrous tissue, which provides flexibility to the newborn skull and acts as an active site of bone formation during skull development in the first two years of life [4,5]. Sutures persist afterwards in toddlers and young adults, as elastic skull sites until flat bone ossification is complete and skull growth ceases, having reached its final size. The inter-suture mesenchyme represents indeed a unique niche for cranial skeletal stem cells, serving as a transient reservoir of MSC and osteoprogenitors [6]. These MSC play a key role in regulating the skull growth: proliferating cells produce new progenitors to support skull growth while a quiescent portion of the MSC population remains in the centre to preserve suture patency and to modulate calvarial bone growth [7]. The osteogenic fate of MSC residing in the suture is driven by the interplay of a complex signalling (including WNT, BMP, FGF and HH pathways as main players) [7,8,9,10] that govern the homeostasis of the calvarial osteogenic niche, essential for calvarial morphogenesis [3,6,10,11]. The disruption of the gene expression pattern and/or of the signalling inside the niche could affect the balance among quiescence–proliferation–differentiation–apoptosis within the suture mesenchyme, causing abnormalities in skull development.

The premature ossification of skull sutures causes craniosynostosis (CS), the second most common congenital craniofacial defect, affecting 1 in ~2500 live births [12,13]. Individuals with CS develop abnormal skull shapes due to premature fusion of one or more cranial sutures, leading to variable degrees of craniofacial dysmorphisms, along with possible increase of intracranial pressure and, in the most severe cases, neurodevelopmental delay. CS occurs in 85% of cases as an isolated and sporadic (i.e., nonfamilial) disorder, in which the abnormal suture fusion apparently results from a developmental defect that directly targets the affected suture(s), without affecting the rest of the body, being hence classified as nonsyndromic craniosynostosis (NCS). NCS is considered a multifactorial disorder, in which gene–gene and/or gene–environment interaction effects are plausibly involved, although their aetiopathogenesis is still largely unclear [8,14,15,16,17]. The complex nature of craniosynostosis is reflected by the difficulty in obtaining univocal therapeutic protocols. To date, the only available therapeutic approach is based on single or repeated reconstructive surgeries, depending on the complexity of the CS and the presence of associated co-morbidities and/or the occurrence of unsatisfying or adverse surgical outcomes [18,19,20,21]. Consequently, understanding the human postnatal craniofacial development and growth and its underlying molecular control is critical for developing adjuvant and possibly personalised therapeutic strategies.

**Figure 1:**
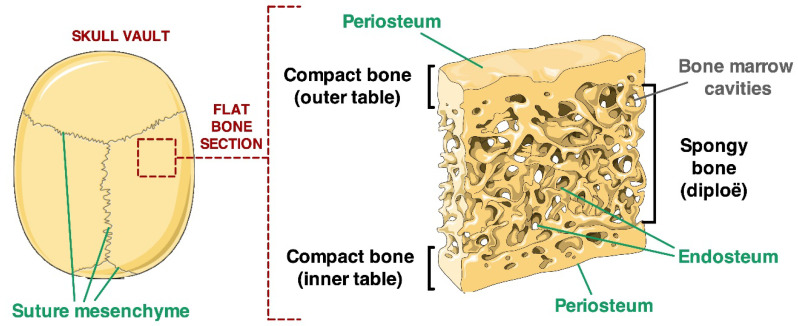
Visual description of the architecture of calvarial flat bone. Two sheets (outer and inner tables) of compact bone enclose a layer of spongy bone (diploë). Green arrows highlight the endosteal, periosteal and inter-suture locations of MSC niches (figure modified from https://smart.servier.com/).

Once their development is completed, craniofacial bones are formed by two layers of compact bone (inner and outer tables) that enclose a sheet of spongy bone (diploe), housing bone marrow (BM) cavities. In this mature bone structure, osteogenic niches are located, as for other bones, in the cambium layer of the periosteum and in the endosteum, which forms the lining of bone marrow cavity walls [6]. The endosteal osteogenic niche is believed to include osteoblasts, endothelial cells, glial cells, vascular pericytes, adipocytes, fibroblasts and MSC [22] The osteogenic and haematopoietic stem cell niches are hence functionally related within the BM environment of the diploe, as MSC support, and regulate the homing of haematopoietic stem cells (HSC), and HSC provide osteoclast precursors that combine with osteogenic lineage’s cells to form the bone structure [23]. Therefore, altogether the calvarial bones’ niches include at least three distinct domains: the endosteal and periosteal domains, which are necessary to accomplish the skull remodelling throughout life, and the transient suture mesenchyme domain that disappear as suture ossify in adult life [6] (Figure 1).

The typical osteogenic stem cell niche has been mostly studied in long bones of the appendicular skeleton, developing through endochondral ossification, while limited information is available about the organisation and homeostasis of the osteogenic niche residing in flat bones, particularly for those residing in the human craniofacial skeleton. A univocal definition of MSC populations and of the differences among different skeletal niches has been long neglected and is yet pending, owing to the inherent cellular heterogeneity, to the different bone developmental path occurring throughout the skeleton, and to the difficulties in studying suitable human flat bone tissues. Recent studies have indeed demonstrated that MSC have distinctive features depending on their in vivo location [24,25].

An in-depth functional characterisation of the osteogenic stem cell niche composition and their involvement in skeletal development was provided by Chan and colleagues, who studied limb bones and bone marrow tissues of foetal, neonatal and adult mice [26]. This study allowed identifying four functionally distinct cell fractions: a CD45+ haematopoietic fraction, a CD45-Tie2 (angiopoietin receptor)+ alpha V integrin (alphaV)+ population that concurrently generates adipocytes and vessels, a CD45-Tie2-alphaV- fraction that does not appear to produce donor-engrafted tissue and a CD45-Tie2-alphaV+ population that, through endochondral ossification, forms bone endowed with bone marrow cavities [26]. 

Calvarial stem cell research has been largely driven by the identification of specific markers expressed by resident populations. In recent years, three distinct cell populations were identified, based on the lineage-specific expression of selected marker genes (namely, GLI1, AXIN2 and THY1), within the sutural mesenchyme of murine models, and proposed as major calvarial skeletal stem cells, or subsets of it [27]. 

GLI1 (GLI Family Zinc Finger 1), a transcriptional key effector of Hedgehog (HH), has been recently proposed as the main marker for the mesenchymal stem cell population in mice, responsible for adult craniofacial bone growth and development [11]. The AXIN2 protein acts as a negative regulator of the Wnt signalling and has been previously implicated in murine calvarial morphogenesis [28]. More recently, the presence of an AXIN2-expressing stem cell population was demonstrated in murine calvarial bones [29]. AXIN2+ cells proved to have long-term self-renewing clonal expansion and differentiation capabilities during calvarial development, suggesting that AXIN2 should be a suitable specific marker for calvarial bone stem cells [29]. Finally, PRX1 (Paired Related Homeobox 1) is a DNA-binding protein expressed in mesodermal tissues, acting as a transcriptional co-activator, implicated in the maintenance of cell fates within the craniofacial mesenchyme. PRX1-expressing cells were shown to reside exclusively in the calvarial suture niche and to decrease in number with age [4]. Interestingly, PRX1 expression seemed to be involved in differentiation of early progenitors into committed osteoblasts, suggesting that this transcription factor could specify the stem cell population in calvarial bones [30]. Taken together, all available data are extremely heterogeneous and not clearly reproducible, hence a calvarial stem cell population is yet to be defined [4,11,29,30].

Moreover, the data available thus far are derived exclusively from studies performed in mice, whose skull bone structure is extremely thin and less layered than in humans. The structure and control of the human calvarial osteogenic niche has not been studied, to date, highlighting a gap of knowledge in the definition of adult stem cells responsible for calvarial bone formation and endogenous regeneration properties.

Our group previously isolated and characterised a population of human multipotent MSC-like cells from suture tissues of patients undergoing surgery for treating NCS [31]. Cells isolated from fused sutures showed a higher osteogenic potential, compared with cells isolated from patient-matched unfused sutures, owing to the constitutive activation of the BMP-dependent signalling [31] and the alteration of the GLI1 expression pattern and related primary cilium signalling [32].

The aim of the present study was to characterise the calvarial MSC and the corresponding niche using NCS patients’ specimens as a model to identify the human craniofacial bone stem cell population. By comparing tissues and cells of fused-versus-unfused sutures of patients, we also aimed to shed light on the pathophysiology of the disease, hence to identify potential cellular and molecular targets to be translated into non-invasive/adjuvant therapeutic strategies. To this aim, the expression of selected markers was analysed in NCS patients’ calvarial bone specimens and in the MSC isolated thereof. Our results indicate the presence of an identifiable calvarial MSC population expressing a specific molecular profile that is dysregulated in fused suture sites, showing abnormal osteogenic differentiation properties. In particular, our data provide evidence that GLI1- and AXIN2-expressing cells reasonably represent the osteogenic stem cells within the human calvarial bone niche.

## 2. Results

### 2.1. Localisation of Calvarial Stem Cell Markers in Suture Tissue Samples 

The expression pattern of AXIN2 and GLI1 in calvarial bone specimens was analysed by immunofluorescence in unfused- and fused-suture tissue samples derived from sagittal NCS patients. In particular, the two markers were tested in a double staining with either the Ki-67 proliferation marker or the THY1 MSC surface antigen, with the aim to assess the presence of a proliferating MSC subpopulation positive also for AXIN2 and GLI1.

**Figure 2:**
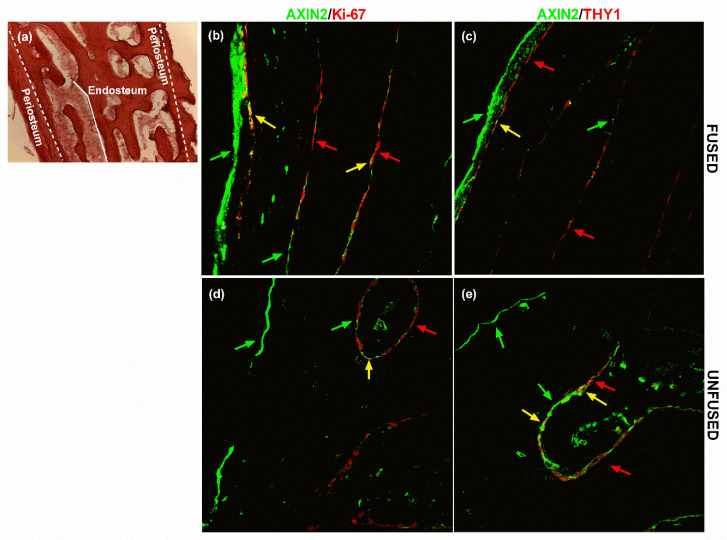
AXIN2 and THY1 expression in calvarial bone. (**a**) The haematoxylin/eosin staining displays the histological structure of a cross-section of the flat calvarial bone, showing the periosteum and endosteum location. Localisation of AXIN2/Ki-67 and AXIN2/THY1 expression in (**b**,**c**) fused and (**d**,**e**) unfused suture tissue sections, assessed by immunofluorescence. Green arrows highlight AXIN2+ cells, red arrows point out (**b**,**d**) Ki-67+ cells and (**c**,**e**) THY1+ cells. Yellow arrows indicate AXIN2+/THY1+ and AXIN2+/Ki-67+ cells. Pictures are representative of 90 microscopic fields from nine slides (technical replicates) at 20× magnification of three patients (biological replicates) (see Methods for details).

**Figure 3:**
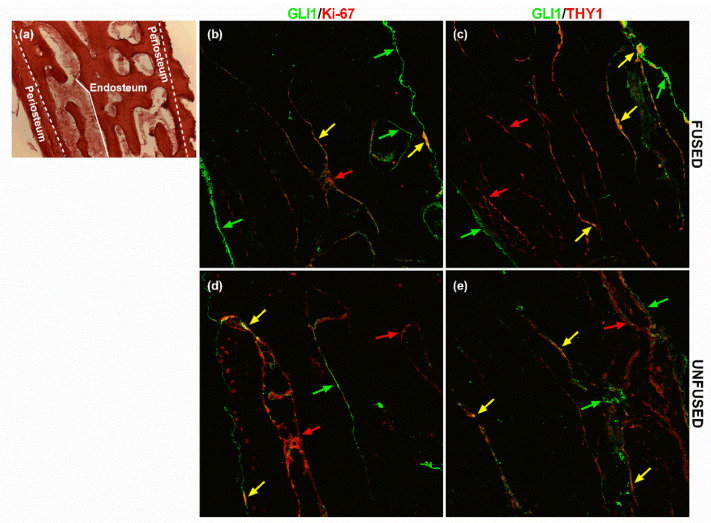
GLI1 and THY1 expression in calvarial bone. (**a**) The haematoxylin/eosin staining displays the histological structure of a cross-section of the flat calvarial bone, showing the periosteum and endosteum location. Localisation of GLI1/Ki-67 and GLI1/THY1 in (**b**,**c**) fused and (**d**,**e**) unfused suture tissue sections, assessed by immunofluorescence. Green arrows highlight cells GLI1+, red arrows point out (**b**,**d**) Ki-67+ cells and (**c**,**e**) THY1+ cells. Yellow arrows indicate GLI1+/THY1+ and GLI1+/Ki-67+ cells. The haematoxylin/eosin staining displays the histological structure of the flat calvarial bone. Pictures are representative of 90 microscopic fields from nine slides (technical replicates) at 20× magnification of three patients (biological replicates) (see Methods for details).

AXIN2-related fluorescence was intensely visible on the periosteal lining and discontinuously expressed on the endosteal side of spongy bone’s trabeculae (Figure 2). Ki-67 was prevalently expressed along the endosteal lining of the diploic trabeculae, with spread spots also visible on the inner periosteal layer on the outer compact bone table (Figure 2b,c and Figure 3b,c). THY1 was scarcely expressed on both endosteal and periosteal sites (Figure 2d,e and Figure 3d,e). 

Conversely, GLI1 expression was visible along the periosteal layer of both the outer and the inner tables and on the endosteal surfaces of the diploic trabeculae (Figure 3).

The double staining allowed observing a subpopulation of AXIN2+/THY1+ and AXIN2+/Ki-67+ cells mostly along the endosteal lining of the trabeculae (Figure 2). As for AXIN2, GLI1 +/Ki-67+ co-expression appeared visible mostly on the endosteal side in the trabecular bone (Figure 3). Above all, a subpopulation of GLI1+/THY1+ cells was present in the endosteal side and along the trabeculae’s margins (Figure 3). In particular, our analysis also showed that there was no remarkable difference in the colocalisation of GLI1 and THY1 between fused and unfused tissue sections (Figure 3).

### 2.2. Expression of Calvarial Stem Cell Markers in Calvarial Mesenchymal Stromal Cells (CMSC)

**Figure 4:**
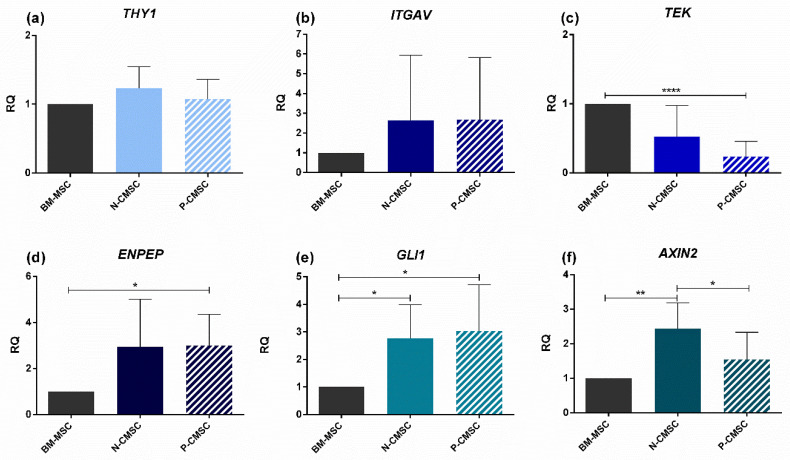
Comparative expression profiling of marker genes in iliac crest BM-MSC and suture cells CMSC. Transcript levels of: (**a**) *THY1*; (**b**) *ITGAV*; (**c**) *TEK*; (**d**) *ENPEP*; (**e**) *GLI1*; and (**f**) *AXIN2*, evaluated by qPCR in N- and P-CMSC, compared with BM-MSC. RQ, Relative Quantity (see Methods for details). * *p* < 0.05; ** *p* < 0.01; **** *p* < 0.0001.

The expression of the selected marker genes *THY1*, *ITGAV*, *TEK*, *ENPEP*, *GLI1* and *AXIN2* was analysed in calvarial MSC isolated both from physiologically patent sutures (termed “Normal”, N-CMSC) and from prematurely fused sutures (termed “Pathologic”, P-CMSC), using bone marrow MSC (BM-MSC) isolated from the iliac crest and grown in standard culture condition as “gold standard” osteogenic MSC controls. The expression of *THY1* and *ITGAV* was comparable among the three tested cell types (Figure 4a,b), whereas the expression of *TEK* showed a reduced trend in CMSC compared with BM-MSC, although the differential expression levels reached statistical significance only in P-CMSC (Figure 4c). The expression of *ENPEP* showed an inverted trend, as it was significantly upregulated in P-CMSC over BM-MSC, with an increased trend also in N-CMSC over BM-MSC (no statistical significance; Figure 4d). GLI1 expression was significantly overexpressed over BM-MSC in both N- and P-CMSC (Figure 4e). Finally, AXIN2 was significantly overexpressed in N-CMSC compared with BM-MSC (Figure 4f). Interestingly, the expression of AXIN2 resulted significantly lower in P-CMSC over N-CMSC (Figure 4f).

### 2.3. Expression of Calvarial Stem Cell Markers during Osteogenic Induction

**Figure 5:**
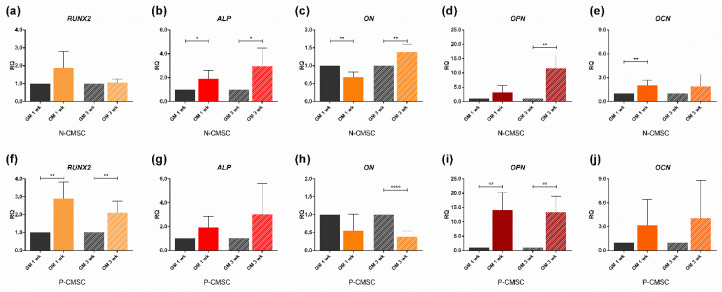
Expression profiles of osteo-specific genes. Transcript levels of: (**a**,**f**) *RUNX2*; (**b**,**g**) *ALP*; (**c**,**h**) *ON*; (**d**,**i**) *OPN*; and (**e**,**j**) *OCN*, evaluated by qPCR in N-CMSC and P-CMSC, respectively, cultured with osteogenic medium (OM) for one (1 wk) and three weeks (3 wk). Cells cultured in standard growth medium (GM) were tested as controls at each time point. RQ, Relative Quantity (see Methods for details). * *p* < 0.05; ** *p* < 0.01; **** *p* < 0.0001.

**Figure 6:**
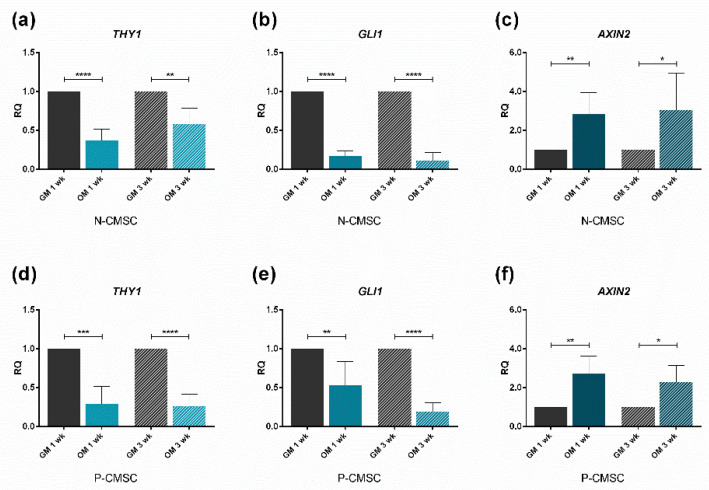
Expression profiles of putative niche marker genes. Transcript levels of: (**a**,**d**) *THY1*; (**b**,**e**) *GLI1*; and (**c**,**f**) *AXIN2*, evaluated by qPCR in N-CMSC and P-CMSC, respectively, cultured with osteogenic medium (OM) for one (1 wk) and three weeks (3 wk). Cells cultured in standard growth medium (GM) were tested as controls at each time point. RQ, Relative Quantity (see Methods for details). * *p* < 0.05; ** *p* < 0.01; *** *p* < 0.001; **** *p* < 0.0001.

Both N- and P-CMSC were efficiently induced toward osteogenic induction up to three weeks, as demonstrated by the upregulation of the osteo-specific marker genes *RUNX2*, *ALP, ON, OPN* and *OCN* (Figure 5). The expression of *THY1*, *GLI1* and *AXIN2* were observed in time course. *THY1* and *GLI1* levels were significantly downregulated in committed N- and P-CMSC after both one and three weeks (Figure 6a,b,d,e). In contrast, *AXIN2* expression was comparable between N- and P-CMSC, being considerably upregulated upon osteogenic induction at both tested time points (Figure 6c,f).

**Figure 7:**
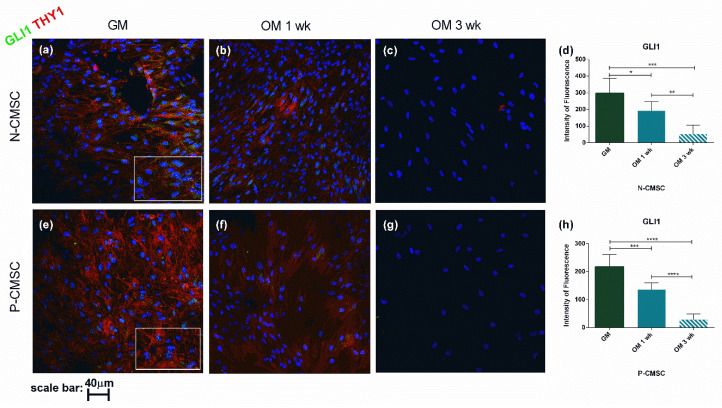
Expression of GLI1 and THY1 in CMSC during osteogenic induction. Markers’ expression analysed by immunofluorescence in (**a**–**c**) N-CMSC and (**e**–**g**) P-CMSC, cultured with osteogenic medium (OM) for one (1 wk) and three weeks (3 wk), compared to cells cultured in growth medium (GM). Nuclei are stained in blue with DAPI. The intensity of fluorescence was quantified by the ImageJ software for both (**d**) N-CMSC and (**h**) P-CMSC. * *p* < 0.05; ** *p* < 0.01; *** *p* < 0.001; **** *p* < 0.0001. Pictures are representative of 30 microscopic fields of three different cell culture samples (see Methods for details).

**Figure 8:**
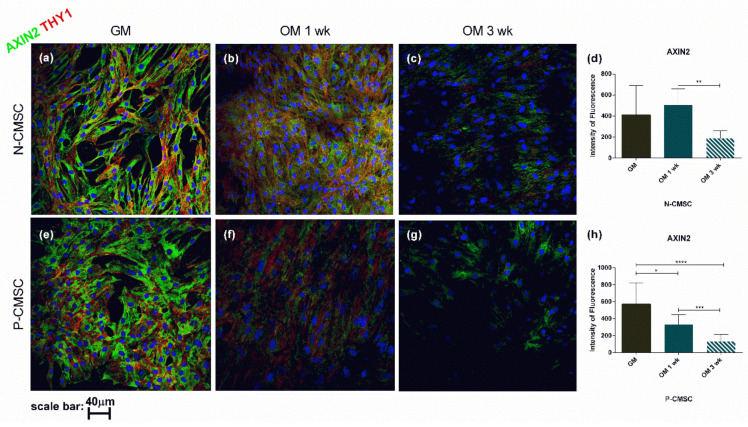
Expression of AXIN2 and THY1 in CMSC during osteogenic induction. Markers’ expression analysed by immunofluorescence in (**a**–**c**) N-CMSC and (**e**–**g**) P-CMSC, cultured with osteogenic medium (OM) for one (1 wk) and three weeks (3 wk), compared to cells cultured in growth medium (GM). Nuclei are stained in blue with DAPI. The intensity of fluorescence has been quantified by the ImageJ software for both (**d**) N-CMSC and (**h**) P-CMSC. * *p* < 0.05; ** *p* < 0.01; *** *p* < 0.001; **** *p* < 0.0001. Pictures are representative of 30 microscopic fields of three different cell culture samples (see Methods for details).

To confirm the gene expression profile, THY1, GLI1 and AXIN2 protein levels were analysed and quantified in situ using immunofluorescence during osteogenic induction. Our results revealed that the expression of all the analysed markers was significantly reduced throughout the in vitro differentiation process (Figure 7 and Figure 8). In particular, GLI1 protein staining was clearly detectable in the cell nuclei and showed a remarkable decrease as early as one week after starting the osteogenic stimulation, being barely detectable after three weeks, in both N- and P-CMSC (Figure 7d,h). Both THY1 and AXIN2 were diffusely expressed in the cytoplasm of cells and tended to decrease during osteogenic induction (see Figure 7a–c,e–g and Figure 8a–c,e–g). The timing of THY1 decrease was comparable in N- and P-CMSC as detected by quantitative estimation of cell staining (data not shown). AXIN2 expression was intense and diffuse in the cell cytoplasm; it appeared apparently unaffected by the osteogenic induction in N-CMSC at one week (Figure 8d), while it significantly decreased in P-CMSC at the same time point (Figure 8h). Thereafter, AXIN2 expression significantly decreased in both N- and P-CMSC at three weeks (Figure 8d,h).

### 2.4. AXIN2 Involvement in the Ossification Process in NCS 

**Figure 9:**
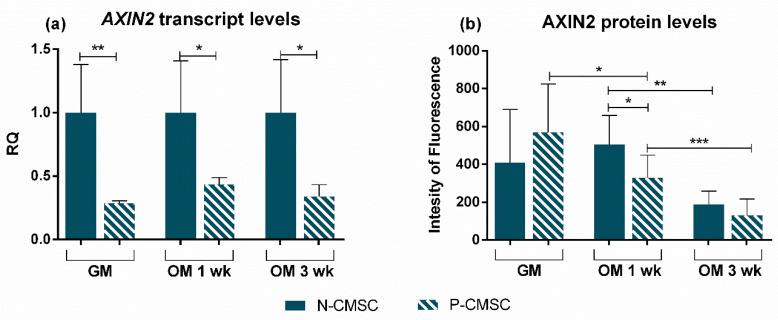
Comparative expression profiling of AXIN2 gene and protein in N-versus-P-CMSC. (**a**) Transcript and (**b**) protein levels of AXIN2 compared in P-CMSC versus N-CMSC, in standard growth conditions (GM) and following one (1 wk) and three weeks (3 wk) of osteogenic commitment (OM). RQ, Relative Quantity (see Methods for details). * *p* < 0.05; ** *p* < 0.01; *** *p* < 0.001.

Since our results show a differential expression of *AXIN2* in P-CMSC and N-CMSC (see Figure 4), we tried to derive possible implications for the pathophysiology of NCS. More in detail, our analysis displayed that *AXIN2* expression was lower in P-CMSC compared with N-CMSC both in standard growth and during osteogenic induction, at both tested time points (Figure 9a). Instead, AXIN2 protein levels were comparable in N- and P-CMSC cultured in growth medium (Figure 9b). Remarkably, AXIN2 expression decreased earlier in P-CMSC compared with N-CMSC upon osteogenic induction (Figure 9b). Taken together, these data indicate that, even though AXIN2 was modulated in response to osteogenic commitment in both N- and P-CMSC, the effect was faster and more evident in cells derived from pathologically fused sutures (Figure 9a,b). 

**Figure 10:**
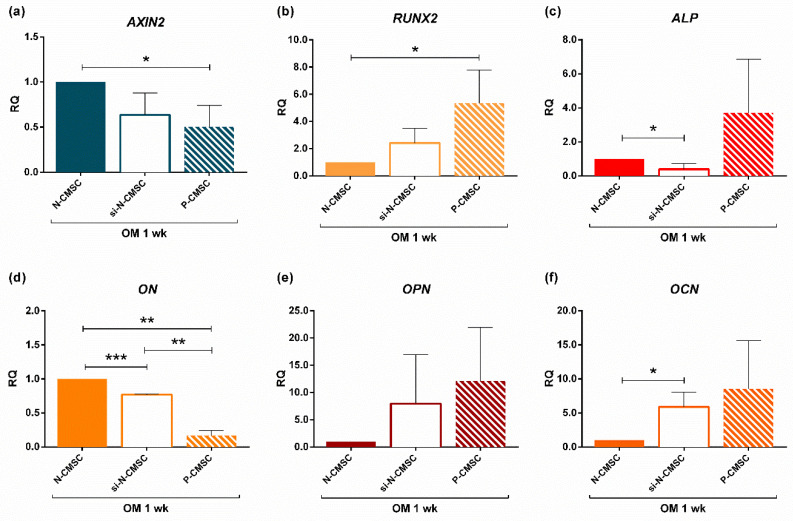
*AXIN2* knockdown results. Expression analysis of: (**a**) *AXIN2*; (**b**) *RUNX2*; (**c**) *ALP*; (**d**) *ON*; (**e**) *OPN;* and (**f**) *OCN*, evaluated by qPCR upon *AXIN2* silencing in N-CMSC (si-N-CMSC) cultured with osteogenic medium for one week (OM 1wk). Untreated N-CMSC were used as controls for both si-N-CMSC and P-CMSC. The graphs show the average values derived from three different experiments on three different patient’s derived cells. RQ, Relative Quantity (see Methods for details). * *p* < 0.05; ** *p* < 0.01; *** *p* < 0.001.

To better clarify this differential modulation of AXIN2 in NCS during the osteogenic process, we knocked down the expression of *AXIN2* in N-CMSC by means of short interfering RNAs (siRNAs), to test whether this could induce an accelerated osteogenic phenotype as seen in P-CMSC in vitro [31]. Gene silencing reduced *AXIN2* expression by 35% (Figure 10a, si-N-CMSC RQ mean: 0.64). The use of higher dosage of siRNAs for *AXIN2* led to a drastic reduction of cell viability after treatment; therefore, the dosage scale was reduced. Interestingly, when *AXIN2* expression in N-CMSC reached levels comparable to that observed in P-CMSC (Figure 10a), this induced the differential expression of selected osteo-specific marker genes upon one week of osteogenic induction (Figure 10b–e). In particular, our analysis showed that the relative quantity of *RUNX2*, *ON*, *OPN* and *OCN* observed in silenced N-CMSC (si-N-CMSC) was significantly different from that observed in untreated N-CMSC, while it followed an expression trend comparable with that observed in P-CMSC (Figure 10b,d–f). Only the expression of ALP showed an opposite trend in si-N-CMSC, being decreased, and in P-CMSC, being upregulated, compared to N-CMSC (Figure 10c). The mock-treatment using exclusively the transfection reagent did not affect the expression of the tested genes at any time point (data not shown).

## 3. Discussion

The idea that a non-haematopoietic multipotent stem cell population resides in the bone marrow (BM) dates back in the early 20th century, with the emerging evidence of the presence of BM stroma-derived cells able to generate tissues of the mesodermal lineages such as bone [33,34]. Since then, the mesenchymal stromal cell (MSC) compartment has been widely characterised within the BM niche found in endochondral bones of the appendicular skeleton of murine models. 

Recent studies attempted the characterisation of the MSC compartment of skull flat bones, describing a calvarial MSC niche responsible for the correct morpho-functional skull development. Thus far, research performed in mice highlighted the presence of specific markers, namely *Gli1*, *Axin2* and *Prx1*, able to efficiently discriminate the MSC population in the cranial bones [4,11,29].

The novelty of the present work resides in that the MSC subpopulation was characterised for the first time in human calvarial bones, and in the assessment of the potential involvement of this bone niche in the pathophysiology of abnormal suture ossification occurring as an isolate developmental defect in nonsyndromic craniosynostosis.

All adult stem cell niche specialised compartments are known to undergo a physiological age-related decline, resulting in the reduced abilities of adult stem cells to sustain quiescence, proliferation capacity and differentiation potential. In craniosynostosis, the calvarial suture stem cells niche is believed to undergo an accelerated and premature senescence, causing a pathological exhaustion of the stem cell reservoir, which ultimately drives the premature ossification of the suture mesenchyme [35]. 

Our study provides the original evidence of the presence of a *GLI1+* MSC subpopulation in the craniofacial bone specimens of patients suffering from nonsyndromic craniosynostosis. GLI1 is a transcription factor that regulates the Hedgehog (HH) transmembrane receptor Patched *PTCH1*, in the “canonical” modulation of the HH pathway [36]. The link between HH and the skeletal development have been extensively investigated [37,38,39]. In particular, HH signalling plays a key role in intramembranous ossification during cranial bone development [40]. Alterations in HH pathway regulation were well documented in several craniofacial abnormalities, including craniosynostosis [40].

Our results demonstrate the presence of a GLI1+ cell subpopulation in the trabecular bone of human cranial tissue samples. The expression of *GLI1* in calvarial MSC isolated from fused and unfused suture tissues of NCS patients resulted significantly higher compared with MSC isolated from hip bone’s bone marrow specimens. These data may point towards defining GLI1 as a putative specific marker for MSC inside the human calvarial niche, representing the osteogenic stem cells supporting the craniofacial bone development inside the human cranial tissue, as already found in murine models [11]. On the other hand, no differences in GLI1 levels were detectable between CMSC derived from fused sutures and those from the unfused one, suggesting that this marker is homogeneously expressed by cells that are not affected by the niche ageing process. Moreover, GLI1 decreased during the in vitro osteogenic induction showing an expression trend that overlaps with that of THY1, a cell surface glycoprotein, widely used as marker of mesenchymal stromal cells, and inversely correlated with the acquisition of the osteogenic committed phenotype. 

In our disease model, we observed that the distribution of AXIN2-expressing cells within the human calvarial niche is comparable between normal and pathological suture specimens, and found in anatomical regions already described in murine models [28,29]. AXIN2 is a negative regulator of the canonical Wnt/β-catenin pathway: *AXIN2* transcription increases upon transduction of a Wnt signal and it provides a negative feedback loop on the pathway, by promoting the degradation of β-catenin [41]. Wnt/β-catenin signalling controls skeletal precursors’ renewal and proliferation and their commitment [10]. AXIN2 is specifically involved in skull development, as it regulates calvarial suture closure [28]. It has been demonstrated that *Axin2* mutant mice present a phenotype resembling craniosynostosis [28,42,43]. In particular, the inactivation of *Axin2* causes an acceleration in osteogenesis, leading to a premature metopic suture closure [28]. Indeed, evidence obtained in murine models showed that *Axin2* is mainly expressed in the osteogenic fronts and in the periosteum, where pre-osteoblasts and osteoblasts are located, and its expression starts decreasing as the suture fusion process starts, being absent in the fused suture [28,29]. 

Our data indicate that *AXIN2* transcript levels are lower in cells isolated from fused sutures than in those from physiologically patent sutures. *AXIN2* expression resulted modulated during osteogenic induction in all tested cells, although both transcript and protein levels were significantly lower in pathologically fused suture-derived cells. Moreover, *AXIN2* knockdown in N-CMSC induces the activation of genes involved in the osteogenic commitment and differentiation of MSC. In particular, our data reveal that the expression of osteo-specific genes induced in N-MSC upon *AXIN2* silencing is comparable to that of observed in P-CMSC during in vitro osteogenic induction. This evidence may confirm that AXIN2 can represent a reliable marker for the stem cell population that undergoes depletion during the premature ossification process occurring in craniosynostosis. Consistently with our results, a de novo loss-of-function mutation in *AXIN2* gene has been recently reported in a patient diagnosed with sagittal craniosynostosis [44].

Nonetheless, our data also show that AXIN2 transcript levels increase, whereas AXIN2 protein levels decrease, in response to in vitro osteogenic differentiation. Further investigation is necessary to interpret this apparent discrepancy, which could be linked to a possible post-transcriptional regulation of AXIN2 (involving RNA processing, trafficking, or decay mechanisms). Such events, to our knowledge, have not been reported in the extant literature.

Finally, the data presented in this study indicate that calvarial MSC isolated from both fused and unfused sutures share the same expression profile for the selected marker genes, upon in vitro isolation through explant culture. This evidence may suggest that the “stemness”-related profile of the cells is not affected by the collection site.

On the other hand, we and other groups previously demonstrated that cells isolated from fused sutures of NCS tend to display a constitutively increased osteogenic activity compared to those isolated from open sutures [17,31,32,45,46,47,48,49,50,51]. This differential osteogenic activity was also observed in fused-versus-unfused suture-derived cells of patients diagnosed with Saethre–Chotzen syndrome, an autosomal dominant CS due to *TWIST1* loss-of-function mutations [52]. This suggests that local functional abnormalities exist at the site of premature suture fusion, even in the presence of documented germline genetic background shared by all cells. 

Taken together, these observations indicate that the tissue-specific niche is inherently preserved (i.e., the same in unfused and fused) in NCS, and the cells maintain the same molecular signature after in vitro isolation, but then local microenvironmental clues provide aberrant stimuli to the cells at the site of the prematurely fused/fusing sutures. Hence, differences in the microenvironment able to modify the niche homeostasis could be responsible for the enhanced in vivo osteogenic differentiation acting at the site of premature suture closure, besides constitutive differences in stem cell properties. This speculative hypothesis is further supported by some recent studies showing that an environmental constraint in vitro induces a specific gene expression profile mimicking that expressed by NCS fused suture-cells [53,54,55].

## 4. Materials and Methods 

All reagents used were purchased from Aurogene (Rome, Italy), if not otherwise specified.

### 4.1. Patient Enrolment and Specimen Collection

We enrolled a sample of 23 patients (mean age 4.7 months; median 4 months; ratio male:female 3.6:1) undergoing cranioplastic surgery for nonsyndromic sagittal craniosynostosis, upon obtaining the written informed consent from their parents.

Suture tissue specimens were collected from both the physiological patent suture (unfused, termed “Normal”, N) and two from the prematurely fused suture (termed “Pathologic”, P) of patients as surgical waste tissues. The sample collected from each suture site was aliquoted into two separate specimens: one placed in culture medium to be used for cell isolation and the other one fixed in formalin and used for immunofluorescence assays. The entire study protocol was designed according to the European Good Clinical Practice guidelines and with the current revision of the Declaration of Helsinki, and was approved by the Ethical Committee of the Università Cattolica del Sacro Cuore, School of Medicine (17 July 2014; protocol NIH/NIDCR 2014, protocol 19029/14).

### 4.2. Calvarial Mesenchymal Stromal Cells (CMSC) Isolation and Culture

CMSC were isolated in primary culture from each tissue specimen (“Normal” N-CMSC; “Pathologic” P-CMSC) harvested from patients and cultured as previously described [31]. Our standard procedure allows selecting adherent cells from the flat bone suture boundary, regardless of the suture ossification status. These cells displayed the phenotype and biological properties of mesenchymal stromal cells (including a CD29+/CD44+/CD105+/CD73+/CD34−/CD31−phenotype and trilineage potential) [31].

Upon reaching confluence, primary cells were detached with trypsin/EDTA and sub-cultivated until the 3rd passage to amplify the cell population and used in further experiments. Mesenchymal stromal cells isolated from human iliac crest bone marrow samples (BM-MSC), representing the gold standard source for MSC isolation, were used as controls. 

### 4.3. Osteogenic Induction

The in vitro osteogenic induction assay was performed as previously described [31]. Briefly, both N-CMSC and P-CMSC at the 3rd–4th culture passage grown until confluence were cultured in osteogenic medium (OM; Dulbecco’s Modified Eagle’s Medium Low Glucose (DMEM), 10% foetal bovine serum (FBS, GIBCO by ThermoFisher Scientific, Waltham, MA, USA), 1% l-glutamine, 1% penicillin–streptomycin, dexamethasone 0.1 µM, β-glycerophosphate (Sigma Aldrich, Saint Louis, MO, USA) 10 µM and ascorbic acid 50 µM, for one (1 wk) and three weeks (3 wk). Medium was changed twice a week. Cells cultured in standard growth medium (DMEM High Glucose, 10% FBS, 1% l-Glutamine and 1% Penicillin-Streptomycin) were used as negative controls for differentiation (GM). 

### 4.4. Gene Expression Analysis

Total RNA was isolated from N- and P-CMSC and BM-MSC at different time points, using Trizol regent (ThermoFisher Scientific, Waltham, MA, USA) following manufacturer’s protocol and subsequently purified using silica membrane spin columns (ReliaPrep RNA™ Cell Miniprep System Promega, San Luis Obispo, CA, USA). RNA was quantified using a UV spectrophotometer (DU 800 Beckman Coulter, Brea, CA, USA). Five hundred nanograms of total RNA were used as template for retrotranscription reaction by GoScript Reverse Kit (Promega, San Luis Obispo, CA, USA) according to the manufacturer’s protocol. cDNA was used as template for Quantitative Real Time PCR analysis (qPCR). The differential expression of *AXIN2*, *GLI1*, *THY1* (also known as CD90), *ITGAV*, *TEK* and *ENPEP* was validated in N- and P-CMSC and in BM-MSC cells. qPCR was also exploited to analyse the expression of selected osteogenic genes as *Runt-related transcription factor 2* (*RUNX2*), *Alkaline phosphatase* (*ALP*), *Osteonectin* (*ON*), *Osteopontin* (*OPN*) and *Osteocalcin* (*OCN*). qPCR was performed using Syber Green master mix (GoTaq qPCR Master Mix Kit, Promega, San Luis Obispo, CA, USA). The sequences of all oligonucleotide primer pairs used for each gene are provided in Table 1. Each sample was analysed in duplicate. The relative expression gene levels were normalised to *Beta-actin* (*ACTB*) levels and quantified according to the ΔΔCt method [56].

### 4.5. Histological Analyses

Suture tissue specimens harvested from patients, were fixed in 4% (*w/v*) formalin in phosphate buffer 0.1 M at pH 7.2 for 72 h at 4 °C. After rinsing with running water, the samples were decalcified in Decalcifier DC2 solution (#11028306 VWR Chemical, Radnor, PA, USA) for 24–96 h (based on sample size) at 4 °C under constant agitation. The tissues were then washed in phosphate buffer saline (PBS) 0.01 M pH 7.4 for 1–2 h at RT, and then cryoprotected in 15% sucrose in PBS overnight at 4 °C. The samples were then embedded in OCT compound, and 10-µm-thick sections were cut on a cryostat (CM 1850, Leica, Wetzlar, Germany) and directly mounted on slides. The slides were used both for haematoxylin and eosin staining and for immunofluorescence analysis. In particular, for histological analysis, the sections were incubated with haematoxylin (according to Mayer, 05-06002/L Bio-Optica, Milan, Italy) for 5 min, washed in running water for 5 min, and then stained with eosin (05-10002/L Bio-Optica, Milan, Italy) for 1 min. Finally, the sections were dehydrated through a series of graded alcohols, clarified in xylene and cover-slipped with Eukitt mounting medium (03989 Sigma Aldrich, Saint Louis, MO, USA). The slides were then examined using a Zeiss Axiophot microscope and the images were captured with an AxioCam 503 colour camera (Zeiss, Jena, Germany) coupled to the microscope.

### 4.6. Immunofluorescence Analysis

Immunofluorescence analysis was performed both on tissue sections and on formalin-fixed CMSC cultures to visualise and quantify the expression of specific-lineage markers during osteogenic induction. In particular, we analysed the expression of Ki-67, THY1, GLI1 and AXIN2. Cells were first permeabilised with 0.3% Triton X-100 for 10 min. Then, both cells and tissue sections were incubated with 3% bovine serum albumin (BSA) in PBS and 3% normal horse serum (NHS)/0.3% Triton X-100 in PBS for 60 min at RT to block nonspecific binding sites. Subsequently, co-immunostainings for AXIN2 or GLI1 with Ki-67 or THY1, respectively, were obtained using the following primary antibodies: mouse anti-THY1 (#MA5-16671 ThermoFisher Scientific, Waltham, MA, USA, 1:100/1:200 respectively for tissue sections and cells), mouse anti-Ki-67 (only for tissue sections) (#ab8191 Abcam, Cambridge, UK, 1.100), rabbit anti-AXIN2 (#PA5-21093 ThermoFisher Scientific, Waltham, MA, USA, 1:100 and 1:500, respectively, for tissue sections and cells) and rabbit anti-GLI1 (#ab49314 Abcam, Cambridge, UK, 1:100 and 1:200, respectively, for tissue sections and cells). After incubating with primary antibodies overnight at 4 °C and washing three times in PBS, the appropriate goat secondary antibodies were added: Alexa Fluor 488 anti-mouse for THY1 and Ki-67 (#305-545-045 Jackson Immunoresearch Laboratories, West Grove, PA, USA, 1:200) or Cy3 anti-rabbit for Gli1 and AXIN2 (#715-166-150 Jackson Immunoresearch Laboratories West Grove PA USA, 1:200) in PBS for 2 h at RT. After further washing, cells were counterstained with DAPI and analysed using the Nikon confocal laser scanning microscopy system (A1MP+, Nikon, Amsterdam, Netherlands) while tissue sections were cover-slipped with Fluoromount Aqueous Mounting Medium (F4680 Sigma–Aldrich, Saint Louis, MO, USA), examined and photographed under a Zeiss LSM 510 confocal laser scanning microscopy system. At least five fields representing each slide were examined in duplicate for each condition. Three non-consecutive tissue sections from each patient were processed for double label immunofluorescence. The quantification of fluorescence intensity was analysed using ImageJ program (NIH-https://imagej.nih.gov/ij/download.html). To verify and confirm the specificity of the immunolabeling, primary antisera were omitted, and only secondary antibodies were used. No immunoreactivity was detected. 

### 4.7. AXIN2 Silencing in N-CMSC

Three chemically synthesised 27-mer Dicer-Substrate Short Interfering RNAs (DsiRNAs, TriFECTa RNAi Kit #hs.Ri.AXIN 2.13, Integrated DNA Technologies, Coralville, IA, USA) were transfected in N-CMSC to knockdown the expression of AXIN2 gene. To this aim, N-CMSC were plated in 6-well plates at a seeding density of 80,000 cells/well and cultured with standard growth medium. Two days after plating, cells at 70% of confluence were transfected with AXIN2-DsiRNAs using Lipofectamine RNAiMAX Reagent (ThermoFisher Scientific, Waltham, MA, USA), according to the manufacturer’s instructions. To test the efficacy of siRNAs constructs, we tested to different concentrations (i.e., 30 and 20 nM). Since the higher concentration critically affected cell viability, all the experiments were performed to a final concentration of 20 nM. After three days, the medium was replaced with OM to induce the osteogenic differentiation. A second dose of AXIN2-DsiRNAs was transfected when OM was replaced. N-CMSC treated exclusively with lipofectamine served as controls for transfection. After 1 week of osteogenic induction, all samples were lysed for RNA extraction and gene expression analysis by qPCR. P-CMSC cultured in both GM and OM were analysed comparatively.

### 4.8. Statistical Analysis

Data were analysed using GraphPad Prism software version 6.0 (San Diego, CA, USA). Results are presented as means ± standard deviation (SD). Statistical differences between groups were analysed using the unpaired Student’s *t*-test. All statistics were two-tailed and the level of significance was set at *p* < 0.05. 

## 5. Conclusions

Our data provide original evidence that GLI1 and AXIN2 reasonably represent specific stem cell markers for the osteogenic niche of human calvarial bones. These findings may represent the foundation for further studies aimed at implementing advanced therapies for skull bone reconstruction and at studying the pathophysiology of altered stem cell mechanisms occurring in craniofacial inborn defect, such as craniosynostosis. In this context, targeting GLI1- and AXIN2-expressing cells would represent a strategy to modulate the proportion and activity of osteoprogenitor cells, hence the osteogenic niche homeostasis, towards the development of patient-tailored approaches aimed at reducing the increased osteogenic rate at the site of premature suture fusion.

Moreover, using nonsyndromic craniosynostosis as a model disease, this study supports the idea of mechanistic effects of multifactorial aetiology underlying this condition, in which concurring chemical and mechanical *noxae* in the environment can modulate the stem cell fate. 

## Figures and Tables

**Table 1:** Primer pairs for qPCR.

Gene	Strand	Sequence
*ACTB*	FW	ACGGCATCGTCACCAACT
*ACTB*	REV	AACGGCAGAAGAGAGAACCA
*THY1*	FW	TCTCCTCCCAGAACGTCACA
*THY1*	REV	GATGCCCTCACACTTGACCA
*ITGAV*	FW	TGTGGCTGTCGGAGATTTCA
*ITGAV*	REV	TTCCCAAAGTCCTTGCTGCT
*TEK*	FW	TGAACACAGTGGCTGGGATG
*TEK*	REV	GTGTCAATCACGTTTGGGGC
*ENPEP*	FW	TGCCAGTGGCGAAAGAAGAG
*ENPEP*	REV	ACAGCAAAGCACACCAGGTA
*GLI1*	FW	TGCACCGAGGGCCCACTCTT
*GLI1*	REV	AGGGAGCTGGGTGAGGTGCG
*AXIN2*	FW	CCTGGCTCCAGAAGATCACA
*AXIN2*	REV	TCAAGCTCTGAGCCTTCAGC
*RUNX2*	FW	GAACCCAGAAGGCACAGACA
*RUNX2*	REV	GGATGAGGAATGCGCCCTAA
*ALP*	FW	CCGTGGCAACTCTATCTTTGG
*ALP*	REV	GCCATACAGGATGGCAGTGA
*ON*	FW	TGCTCCCCAGGCAAAAGAAG
*ON*	REV	AGGGCTTGCACTTGACCAAA
*OPN*	FW	TGAAACGAGTCAGCTGGATG
*OPN*	REV	GCTCTCATCATTGGCTTTCC
*OCN*	FW	GACTGTGACGAGTTGGCTGA
*OCN*	REV	AGCAGAGCGACACCCTAGAC

FW, forward primer; REV, reverse primer.

## Abbreviations

MSC	Mesenchymal stromal cells
WNT	Wingless-type MMTV integration site family
BMP	Bone morphogenetic protein family
FGF	Fibroblast growth factor family
HH	Hedgehog signalling
CS	Craniosynostosis
NCS	Nonsyndromic craniosynostosis
BM	Bone marrow
HSC	Haematopoietic stem cell
TEK/Tie2	TEK receptor tyrosine kinase
ITGAV/AlphaV	Integrin subunit alpha V
GLI1	GLI Family Zinc Finger 1
THY1	Thy-1 cell surface antigen
PRX1	Paired Related Homeobox 1
ENPEP	Glutamyl aminopeptidase
CMSC	Calvarial mesenchymal stromal cells
N-CMSC	Normal-CMSC
P-CMSC	Pathologic-CMSC
BM-MSC	Bone marrow mesenchymal stromal cells
RUNX2	RUNX family transcription factor 2
ALP	Alkaline phosphatases
ON	Osteonectin
OPN	Osteopontin
OCN	Osteocalcin
OM	Osteogenic medium
GM	Growth medium
RQ	Relative quantity
siRNAs	Short interfering RNAs
si-N-CMSC	Silenced N-CMSC
PTCH1	Patched 1
EDTA	Ethylenediaminetetraacetic Acid
DMEM	Dulbecco’s Modified Eagle’s Medium
FBS	Fetal bovine serum
qPCR	Quantitative Real Time PCR
ACTB	Beta-actin
PBS	Phosphate buffer saline
RT	Room temperature
BSA	Bovine serum albumin
